# PReS-FINAL-2161: Safety and effectiveness of adalimumab in children with polyarticular juvenile idiopathic arthritis aged 2 to <4 years or >=4 years weighing <15 kg

**DOI:** 10.1186/1546-0096-11-S2-P173

**Published:** 2013-12-05

**Authors:** D Kingsbury, P Quartier, V Arora, J Kalabic, H Kupper, N Mozaffarian

**Affiliations:** 1 Randall Children's Hospital at Legacy Emanuel, Portland, OR, USA; 2 Hôpital Necker-Enfants Malades, Paris, France; 3 abbvie Inc., North Chicago, IL, USA; 4 abbvie Deutschland gmbh & Co. KG, Ludwigshafen, Germany

## Introduction

Adalimumab (ADA) is approved for use in moderate to severe juvenile idiopathic arthritis (JIA) in patients (pts) ≥4 years (yrs) old in the US, EU, and Japan. Limited data are available in pts <4 yrs old.

## Objectives

To assess the safety and effectiveness of >1 year of ADA therapy in pts aged 2 to <4 yrs old or ≥4 yrs old weighing <15 kg with moderately to severely active polyarticular JIA

## Methods

JIA pts were treated with ADA 24 mg/m^2 ^(maximum = 20 mg/dose) every other week (wk) +/- methotrexate for a minimum of 24 wks in an ongoing international, multicenter, open-label, phase 3b study until achieving the limit for age (≥4 yrs) and weight (≥15 kg). Adverse events (aes) were summarized for all visits up to 96 wks. Clinical effectiveness endpoints were assessed as observed, and included American College of Rheumatology pediatric (pedacr) 30/50/70/90 responses through wk 60, and JIA outcome parameters (Physician's Global Assessment [phga] and Parent Global Assessment [paga] of Disease Activity, paga of Pain [all 3 on a VAS of 0-100 mm], Disability Index of Childhood Health Assessment Questionnaire [DICHAQ], Active Joint Count [AJC73], Limitation on Passive Motion [LOM69], C-Reactive Protein [CRP], Tender Joint count [TJC], Swollen Joint Count [SJC], and Pain on Passive Motion [POM75]).

## Results

32 pts were randomized; through wk 60, two pts withdrew due to aes (JIA worsening or flare) and 2 withdrew for other reasons. AE incidence rates included: any aes (91%), serious aes (16%), infectious aes (78%), and serious infections (9%). No deaths, malignancies, or opportunistic infections were reported. 90% of pts had achieved pedacr30 at wk 60 (Table 1). High pedacr 50/70/90 response rates were achieved at wk 24 and maintained through wk 60. Statistically significant improvements in other JIA outcomes were also observed at wk 60. Mean change (SD) for these outcomes were: phga [-42.7(28.2)], paga [-34.5 (33.3)], DICHAQ [-0.6 (0.7)], AJC73 [-9.5 (7.5)], LOM69 [-5.5 (8.3)], CRP(mg/dl) [-0.3 (1.8)], TJC75 [-4.5 (5.9)], SJC66 [-8.4 (7.2)], POM75 [-5.9 (5.3)], and paga of Pain [-35.2 (34.4)]. Growth was not adversely impacted by ADA treatment; based on CDC growth standards, at baseline, 50%/53% of pts were in the ≥33rd percentile for height and body mass index, respectively; at wk 60, this had increased to 76%/67%.

**Table 1:** Pedacr Response Over Time

	Week 24 Response Rate N = 30 N (%)	Week 60 Response Rate N = 20 N (%)
Pedacr 30	27 (90.0)	18 (90.0)
Pedacr 50	25 (83.3)	16 (80.0)
Pedacr 70	22 (73.3)	14 (70.0)
Pedacr 90	11 (36.7)	10 (50.0)

## Conclusion

In this very young population with polyarticular JIA, primary clinical trial data revealed that the safety profile and effectiveness of ADA were comparable to that observed in older children with JIA. Moreover, ADA had no adverse impact on height or weight, as data reflected improvement in growth metrics through wk 60 of the study.

## Disclosure of interest

D. Kingsbury Grant/Research Support from: abbvie Inc., P. Quartier Grant/Research Support from: abbvie, Novartis, Chugai-Roche and Pfizer, Consultant for: abbvie, Novartis, Chugai-Roche and Pfizer, V. Arora Shareholder of: abbvie Inc., Employee of: abbvie Inc., J. Kalabic Shareholder of: abbvie Inc., Employee of: abbvie Inc., H. Kupper Shareholder of: abbvie Inc., Employee of: abbvie Inc., N. Mozaffarian Employee of: Former employee of abbvie Inc.

